# Acute Saddle Embolus With Thrombus in Transit: A Case Report and Review of the Literature

**DOI:** 10.7759/cureus.25018

**Published:** 2022-05-15

**Authors:** Andrew V Doodnauth, Claire S Choi, Julian C Dunkley, Elizabeth M Zharovsky, Toan M Nguyen, Noriyuki Murakami

**Affiliations:** 1 Internal Medicine, State University of New York (SUNY) Downstate Medical Center, Brooklyn, USA; 2 Internal Medicine, State University of New York (SUNY) Downstate College of Medicine, Brooklyn, USA; 3 Psychiatry, State University of New York (SUNY) Downstate Medical Center, Brooklyn, USA

**Keywords:** pulmonary embolism, saddle embolus, mortality, clot in transit, left ventricular systolic dysfunction

## Abstract

Pulmonary embolism is a life-threatening medical emergency associated with right ventricular failure. Rarely, it impacts the left ventricle to the point of compromising the left ventricular (LV) ejection fraction. We present a case of a 73-year-old African American male with a medical history pertinent for intravascular large B-cell lymphoma who developed an acute saddle embolus with a “clot-in-transit” and profound LV systolic dysfunction. Our report illustrates how an acute saddle embolus may be associated with LV systolic dysfunction via the “reverse Bernheim effect.” Additionally, the report highlights the significance of a “clot-in-transit” and LV systolic dysfunction, as they both directly correlate with increased risk of mortality.

## Introduction

Pulmonary embolism (PE) is a common, life-threatening medical emergency with a significant mortality rate as high as 30% [1]. Due to the increased platelet activation and hypercoagulation associated with malignancy, the risk is greater in cancer patients with up to 78% increased risk for PE than the general population [2,3], with a higher incidence specifically in hematologic malignancies [4]. PE primarily results in acute right ventricular (RV) failure with hemodynamic collapse secondary to pulmonary bed obstruction and a sudden increase in RV afterload. However, to the best of our knowledge, the current literature reports only a few cases of acute submassive PE-induced left ventricular (LV) systolic dysfunction. Here, we describe a case of an acute submassive saddle embolus with profound LV systolic dysfunction in an elderly African American male with intravascular large B-cell lymphoma (IVLBCL). We highlight the pathophysiology, management, and importance of early recognition and prognosis.

## Case presentation

A 73-year-old African American male was brought into the emergency department (ED) by emergency medical services (EMS) for presumed syncope. The patient’s past medical history was pertinent for hypertension (HTN) and recently diagnosed IVLBCL (stage IV). The patient received his first cycle of R-CHOP chemotherapy (prednisone 10 mg, rituximab 375 mg/m^2^, cyclophosphamide 750 mg/m^2^, doxorubicin 50 mg/m^2^, vincristine 1.4 mg/m^2^) two weeks prior and completed his third dose of intrathecal methotrexate one week before admission.

Per family, the patient had been feeling progressively tired and weak following his recent chemotherapy session, affecting his ability to perform his activities of daily living. They failed to reach him via phone and emergently contacted EMS, who found him unconscious on the apartment floor. Initial vitals were within normal limits, including blood glucose fingerstick. EMS transported the patient to the nearest emergency department.

**Figure 1:**
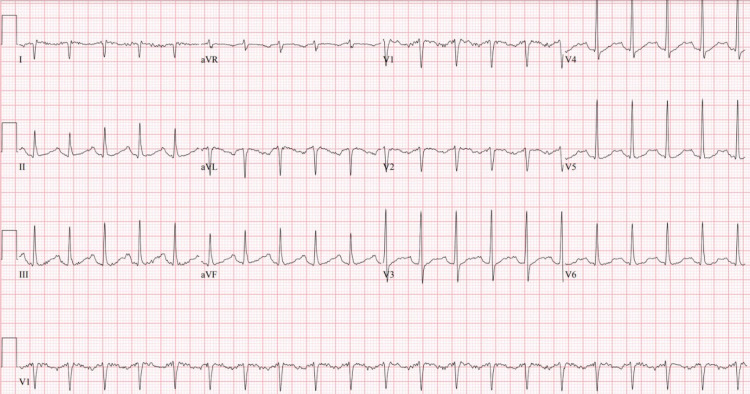
EKG: junctional tachyarrhythmia, left posterior fascicular block, heart rate of 124 beats/minute, and corrected QT of 612 ms.

In the ED, repeat vitals showed a blood pressure of 138/79 mmHg, heart rate of 122 beats/minute, respiratory rate of 18 breaths/minute, S_p_O_2_ at 99% on room air, and a temperature of 101.9°F. The patient was alert, oriented, and in no acute distress. The remainder of the physical examination was unremarkable except for dry oral mucosa. Initial laboratory values were significant for an elevated troponin-I of 0.31 (<0.15 ng/mL) and brain natriuretic peptide (BNP) of 1,044 (<100 pg/mL). The chest radiograph showed no acute process. An electrocardiogram (EKG) showed a junctional tachyarrhythmia and posterior fascicular block with a heart rate of 124 beats/minute (Figure 1).

Differential diagnoses included but were not limited to sepsis, PE, and type 1 and type 2 myocardial infarction. The patient was given 2 L of lactated Ringer's solution and started on vancomycin 1250 mg q12hr, piperacillin-tazobactam 3.375 mg q8hr, and given one dose of 325 mg aspirin.

**Figure 2:**
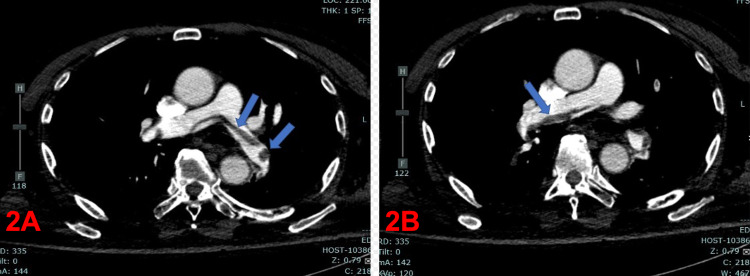
Computed tomography angiography pulmonary embolus series showing saddle embolus. A: Blue arrows showing thrombus occluding the left pulmonary artery. B: Blue arrow showing thrombus occluding the right pulmonary artery.

Serial troponin-I continued to rise to 0.31, 0.52, 0.92, and 0.96 (<0.15 ng/mL). Although the patient's blood pressure remained stable, his tachycardia persisted upon telemetry review with a peak heart rate of 135 beats/minute. Additionally, he became tachypneic, with a respiratory rate trending upwards to 26 breaths/minute. With high clinical suspicion of PE in the setting of known malignancy, computed tomography angiography (CTA) of the chest revealed a saddle embolism with right heart strain (Figure 2). We diagnosed a submassive PE, discontinued antibiotics, and started the patient on full-dose low molecular weight heparin (LMWH).

**Figure 3:**
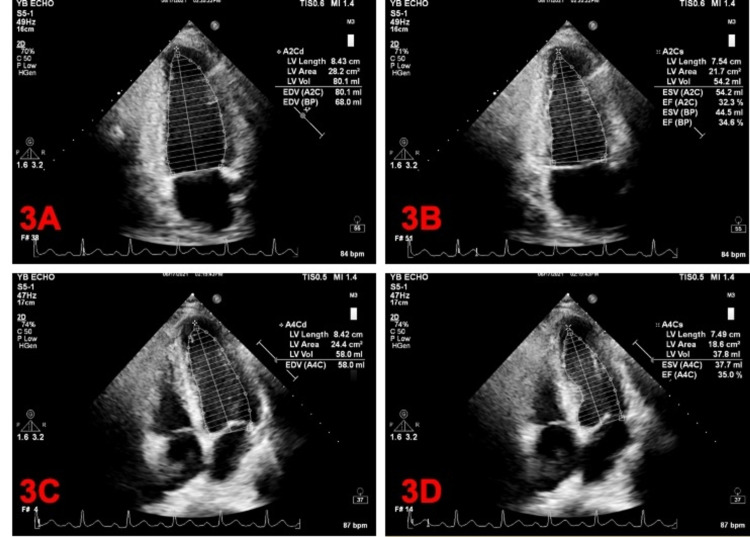
Modified Simpson method. A: Apical two-chamber diastole. B: Apical two-chamber systole. C: Apical four-chamber diastole. D: Apical four-chamber systole. A2C = apical two chamber; A2Cd = apical two chamber diastole; A2Cs = apical two chamber systole; A4Cd = apical four chamber diastole; A4Cs = apical four chamber systole; LV = left ventricular; BP = biplane; EDV = end diastolic volume; EF = ejection fraction; ESV = end systolic volume.

**Figure 4:**
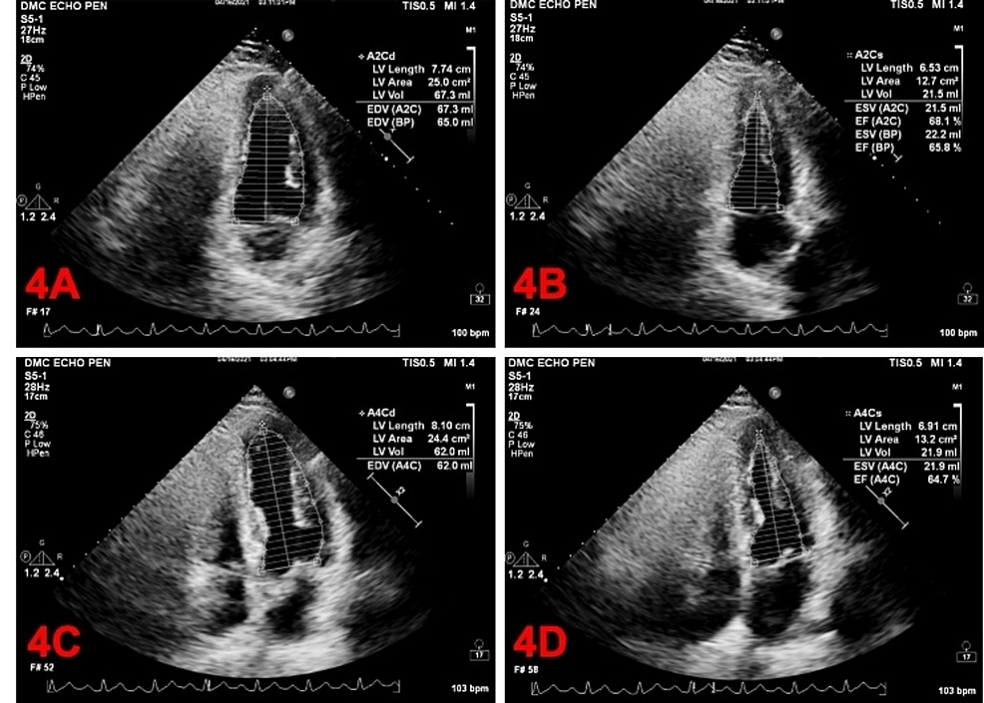
Modified Simpson method. A: Apical two-chamber diastole. B: Apical two-chamber systole. C: Apical four-chamber diastole. D: Apical four-chamber systole. A2C = apical two chamber; A2Cd = apical two chamber diastole; A2Cs = apical two chamber systole; A4Cd = apical four chamber diastole; A4Cs = apical four chamber systole; LV = left ventricular; BP = biplane; EDV = end diastolic volume; EF = ejection fraction; ESV = end systolic volume.

The team appropriately ordered a transthoracic echocardiogram (TTE) to assess the extent and severity of the cardiac injury. We discovered moderate RV dilation. It also showed new LV diffuse hypokinesis with wall thickness within acceptable limits and an ejection fraction estimated at around 30% (Figure 3). Interestingly, TTE performed two months prior showed an ejection fraction of around 60% with no clinically significant valvular or wall motion abnormalities (Figure 4).

An interdisciplinary team, including medicine, oncology, and cardiology, discussed prognosis and goals of care with the patient and their family. Due to new findings on the echocardiogram, consistent with increased mortality, the patient ultimately decided against pursuing further chemotherapy. The team did not advocate for goal-directed medical therapy for the new-onset cardiomyopathy.

The patient remained hemodynamically stable for the remainder of the hospital course with no complaints of shortness of breath or chest pain and no longer required supplemental oxygen. Physical therapy evaluated the patient and recommended sub-acute rehabilitation (SAR). Upon discharge, we transitioned the patient to full-dose apixaban with instructions to follow up with his primary care physician.

## Discussion

PE is a form of venous thromboembolism (VTE) that is relatively common among patients presenting to the ED, affecting nearly 900,000 people in the United States each year, with 60,000-100,000 of these events being fatal [5]. We diagnosed our patient with an acute submassive PE. A saddle PE lodges at the bifurcation of the main pulmonary artery, often extending into the right and left main pulmonary arteries; consistent with our patient’s CTA, and occurs in approximately 3-6% of patients presenting with a PE [6,7]. Although a saddle embolus may have catastrophic complications, retrospective analysis suggests that among those diagnosed with a saddle embolus, only 22% are hemodynamically unstable, with an associated mortality of 5% [6,7].

Our patient recently completed R-CHOP chemotherapy (prednisone 10 mg, rituximab 375 mg/m^2^, cyclophosphamide 750 mg/m^2^, doxorubicin 50 mg/m^2^, vincristine 1.4 mg/m^2^) two weeks prior to presentation. New findings of LV systolic dysfunction on TTE were originally thought to be from doxorubicin, a known culprit to cause cardiotoxicity, which may occur acutely within two to three days of its administration; an incidence of approximately 11% [8,9]. Typically, patients acutely present with chest pain due to myopericarditis and/or palpitations due to arrhythmia. However, acute LV failure is a rare manifestation of acute cardiotoxicity. The incidence of chronic doxorubicin cardiotoxicity is much lower, with an estimated incidence of about 1.7%, usually evident within 30 days of administration of its last dose [10]. The incidence of doxorubicin cardiomyopathy is primarily related to cumulative dosage. The incidence is about 4% when the dose of doxorubicin is 500-550 mg/m^2^, 18% when the dose is 551-600 mg/m^2^, and 36% when the dose exceeds 600 mg/m^2^ [11]. Our patient only received a total dosage of approximately 200 mg/m^2^.

IVLBCL is a rare, clinically aggressive subtype of lymphoma characterized by the predominant growth of large cells within the lumen of blood vessels, particularly capillaries and post-capillary venules resulting in diffuse thrombosis. Given the highly aggressive nature of this disease, approximately 60% of IVLBCL patients present with stage IV disease with involvement in multiple organs including the integumentary, central nervous system (CNS), bone marrow, liver, and spleen [12]. While vascular obstruction due to IVLBCL most commonly occurs in the CNS and the skin, there have been a few reported cases of cardiac involvement. Kadoya et al. [13] and Bauer et al. [14] presented evidence of large B-cell lymphoma cell infiltration into the myocardial microvessels at autopsy after death from myocardial infarction. A case of rapidly progressive RV heart failure due to lymphomatous obstruction of pulmonary vessels from IVLBCL has been reported as well [15]. It is well known that patients with heart failure have an increased risk of developing PE due to low cardiac output and hemostasis abnormalities including platelet and endothelial dysfunction [16]. In this aspect, the acute LV systolic dysfunction may be due to the invasion of lymphoma cells into the myocardial interstitium and small coronary vessels with PE as a subsequent complication of his acute heart failure. Nevertheless, per chart review, there was no prior documentation from the outside hospital to convey a diagnosis of heart failure before presenting at our ED. In addition, our patient presented with no chest pain or any anginal equivalent, and our initial EKG was not consistent with a type 1 ischemic event causing decompensation. As a result, our working diagnosis of acute PE as the likely cause of the newly identified LV systolic dysfunction remained atop the differential diagnosis.

**Figure 5:**
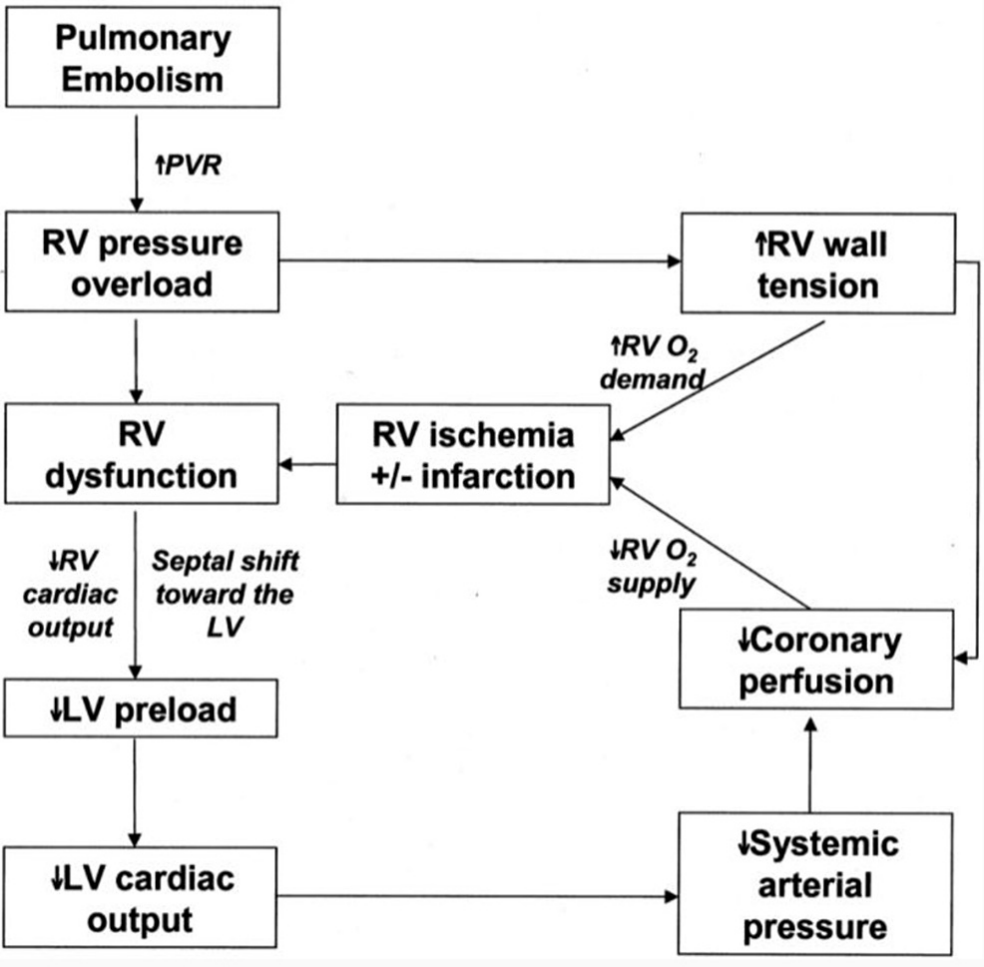
The pathophysiology of acute pulmonary embolism causing left ventricular systolic dysfunction. PE = pulmonary embolism; PVR = pulmonary vascular resistance; RV = right ventricle; LV = left ventricle.

It appears that LV dysfunction in the presence of acute PE portends a higher risk of mortality [5]. LV systolic dysfunction commonly results from decreased RV output [16]. Acute PE increases pulmonary vascular resistance secondary to direct physical obstruction of the vascular bed, hypoxemia, and vasoconstriction within the pulmonary arterial system [17] (Figure 5). The acute rise in afterload causes RV dilation, flattening the interventricular septum and altering LV diastolic compliance and interventricular interdependence via the so-called “reverse Bernheim effect” [18-20]. Overall, this reduces LV preload and stroke volume, compromising LV ejection fraction [21].

**Figure 6:**
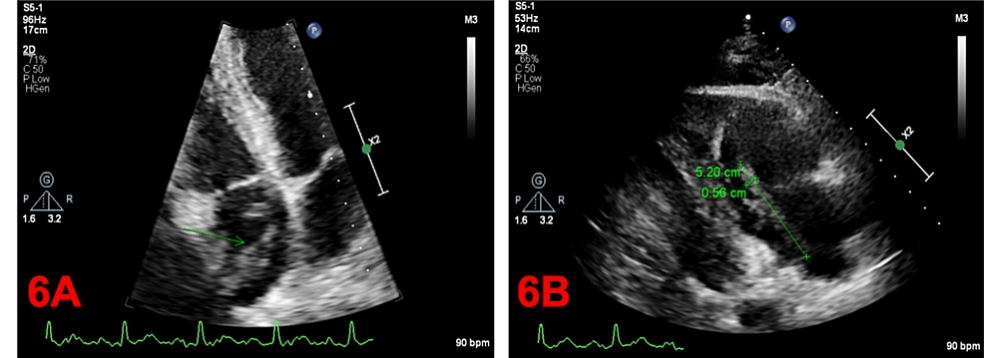
A: The right atrium is mildly dilated with large possibly multilobed elongated mobile mass, originating in the posterior atrium (exact attachment not clear). B: Presumable thrombus prolapsing across the tricuspid valve, cannot exclude another mass lesion.

TTE identified a clot-in-transit, which is associated with high mortality (up to 40%) (Figure 6) [6,7]. The development of intracardiac thrombi stems from Virchow’s triad: stasis, endothelial injury, and hypercoagulable state. The standard of care for intracardiac thrombus is a treatment with a vitamin K antagonist (VKA). However, observational studies shape the current guidelines [22]. Warfarin poses many challenges to both clinicians and patients because of its slow onset of action, bleeding complications, narrow therapeutic window requiring frequent monitoring, and interactions with various foods and drugs, making direct oral anticoagulants (DOACs) a very appealing alternative [23]. To date, there are no large randomized trials that support the use of DOACs in the management of intracardiac thrombosis. Ultimately, we decided to transition our patient from LMWH to apixaban due to multiple published articles providing evidence on the efficacy of DOACs in treating intracardiac thrombosis of the left ventricle [24].

Early recognition remains essential for acute PE, as it is the third most common acute cardiovascular disease after myocardial infarction and stroke [25]. The Pulmonary Embolism Severity Index (PESI) is a well-established predictive model of mortality for patients diagnosed with acute PE [26]. Unfortunately, a flaw in the model is that it does not include the echocardiographic data, resulting in an underestimation of overall mortality. As shown in our patient, the calculator tabulated a score of 156 points, consistent with class V (>125 points) and a 10.0-24.5% 30-day mortality risk; when in actuality, his mortality was significantly higher.

## Conclusions

Prompt recognition of an acute PE and its complications remains critical, especially in patients with underlying malignancy. If left untreated, there is significant morbidity and mortality. Physicians should be aware of poor prognosticators, i.e., “clot-in-transit” and LV systolic dysfunction. Early administration of full-dose anticoagulation for an acute PE remains the hallmark of treatment.

## References

[1] Bĕlohlávek J, Dytrych V, Linhart A (2013). Pulmonary embolism, part I: epidemiology, risk factors and risk stratification, pathophysiology, clinical presentation, diagnosis and nonthrombotic pulmonary embolism. Exp Clin Cardiol.

[2] Reynolds MW, Shibata A, Zhao S, Jones N, Fahrbach K, Goodnough LT (2008). Impact of clinical trial design and execution-related factors on incidence of thromboembolic events in cancer patients: a systematic review and meta-analysis. Curr Med Res Opin.

[3] Stein PD, Matta F (2010). Epidemiology and incidence: the scope of the problem and risk factors for development of venous thromboembolism. Clin Chest Med.

[4] Blom JW, Doggen CJ, Osanto S, Rosendaal FR (2005). Malignancies, prothrombotic mutations, and the risk of venous thrombosis. JAMA.

[5] Liteplo AS, Huang CK, Zheng H (2021). Left ventricular dysfunction correlates with mortality in pulmonary embolism. J Emerg Med.

[6] Ryu JH, Pellikka PA, Froehling DA, Peters SG, Aughenbaugh GL (2007). Saddle pulmonary embolism diagnosed by CT angiography: frequency, clinical features and outcome. Respir Med.

[7] Sardi A, Gluskin J, Guttentag A, Kotler MN, Braitman LE, Lippmann M (2011). Saddle pulmonary embolism: is it as bad as it looks? A community hospital experience. Crit Care Med.

[8] Takemura G, Fujiwara H (2007). Doxorubicin-induced cardiomyopathy from the cardiotoxic mechanisms to management. Prog Cardiovasc Dis.

[9] Swain SM, Whaley FS, Ewer MS (2003). Congestive heart failure in patients treated with doxorubicin: a retrospective analysis of three trials. Cancer.

[10] Von Hoff DD, Layard MW, Basa P, Davis HL Jr, Von Hoff AL, Rozencweig M, Muggia FM (1979). Risk factors for doxorubicin-induced congestive heart failure. Ann Intern Med.

[11] Lefrak EA, Pitha J, Rosenheim S, Gottlieb JA (1973). A clinicopathologic analysis of adriamycin cardiotoxicity. Cancer.

[12] Ponzoni M, Campo E, Nakamura S (2018). Intravascular large B-cell lymphoma: a chameleon with multiple faces and many masks. Blood.

[13] Kadoya Y, Nagaoka S, Kanatani M (2021). Imaging findings of a case of intravascular large B-cell lymphoma with cardiac involvement. Radiol Case Rep.

[14] Bauer A, Perras B, Sufke S, Horny HP, Kreft B (2005). Myocardial infarction as an uncommon clinical manifestation of intravascular large cell lymphoma. Acta Cardiol.

[15] Share M, Giannini G, Kim S, Singh S (2019). Intravascular B-cell lymphoma: case report of a rare cause of pulmonary arterial hypertension. Eur Heart J Case Rep.

[16] Piazza G, Goldhaber SZ (2005). The acutely decompensated right ventricle: pathways for diagnosis and management. Chest.

[17] Alpert JS, Francis GS, Vieweg WV, Thompson SI, Stanton KC, Hagan AD (1977). Left ventricular function in massive pulmonary embolism. Chest.

[18] Taylor RR, Covell JW, Sonnenblick EH, Ross J Jr (1967). Dependence of ventricular distensibility on filling of the opposite ventricle. Am J Physiol.

[19] Salel A, Mason DT, Amsterda EA, Zelis R (1971). Depression of left ventricular contractility in primary right ventricular overload: the "reverse Bernheim phenomenon". Circulation.

[20] Dexter L (1956). Atrial septal defect. Br Heart J.

[21] Jaff MR, McMurtry MS, Archer SL (2011). Management of massive and submassive pulmonary embolism, iliofemoral deep vein thrombosis, and chronic thromboembolic pulmonary hypertension: a scientific statement from the American Heart Association. Circulation.

[22] Vaitkus PT, Barnathan ES (1993). Embolic potential, prevention and management of mural thrombus complicating anterior myocardial infarction: a meta-analysis. J Am Coll Cardiol.

[23] Savelieva I, Camm AJ (2014). Practical considerations for using novel oral anticoagulants in patients with atrial fibrillation. Clin Cardiol.

[24] Shokr M, Ahmed A, Abubakar H, Sayedahmad Z, Rashed A, Afonso L, Cardozo S (2019). Use of direct oral anticoagulants in the treatment of left ventricular thrombi: a tertiary center experience and review of the literature. Clin Case Rep.

[25] Giuntini C, Di Ricco G, Marini C, Melillo E, Palla A (1995). Pulmonary embolism: epidemiology. Chest.

[26] Aujesky D, Obrosky DS, Stone RA (2005). Derivation and validation of a prognostic model for pulmonary embolism. Am J Respir Crit Care Med.

